# Changes in a Comprehensive Profile of Saliva Analytes in Fattening Pigs during a Complete Productive Cycle: A Longitudinal Study

**DOI:** 10.3390/ani12141865

**Published:** 2022-07-21

**Authors:** Alba Ortín-Bustillo, Damián Escribano, Marina López-Arjona, María Botia, Pablo Fuentes, Silvia Martínez-Miró, Camila P. Rubio, Edgar García-Manzanilla, Lorena Franco-Martínez, Luis Pardo-Marín, José J. Cerón, Pol Llonch, Fernando Tecles

**Affiliations:** 1 Interdisciplinary Laboratory of Clinical Analysis of the University of Murcia (Interlab-UMU), Regional Campus of International Excellence ‘Campus Mare Nostrum’, University of Murcia, Campus de Espinardo s/n, 30100 Murcia, Spain; alba.ortinb@um.es (A.O.-B.); det20165@um.es (D.E.); marina.lopez10@um.es (M.L.-A.); maria.botiag@um.es (M.B.); lorena.franco2@um.es (L.F.-M.); lpm1@um.es (L.P.-M.); jjceron@um.es (J.J.C.); 2 Department of Animal Production, Regional Campus of International Excellence ‘Campus Mare Nostrum’, University of Murcia, Campus de Espinardo s/n, 30100 Murcia, Spain; 3 Cefu S.A., 30840 Murcia, Spain; pablo.fuentes@cefusa.com (P.F.); silviamm@um.es (S.M.-M.); 4 Department of Animal and Food Science, Universitat Autònoma de Barcelona, 08193 Bellaterra, Spain; camila.peres@um.es (C.P.R.); pol.llonch@uab.cat (P.L.); 5 Pig Development Department, Teagasc Grassland Research and Innovation Centre, P61 C996 Fermoy, Ireland; edgar.garciamanzanilla@teagasc.ie; 6 School of Veterinary Medicine, University College Dublin, D04 V1W8 Dublin, Ireland

**Keywords:** saliva, biomarker, fattening pigs, productive phase, gender

## Abstract

**Simple Summary:**

The aim of this study was to evaluate whether a panel of 29 salivary biomarkers of stress, immunity, inflammation, redox homeostasis and other physiological functions can change in healthy fattening pigs when monitoring the different phases of their productive cycle and can be influenced by various sources of variations such as gender and performance parameters. Several analytes showed changes due to the productive cycle, with a majority of the analytes showing higher values at lactation and at the beginning of nursery. Additionally, differences were seen due to sex. These differences can be related in some cases with performance parameters and should be taken into consideration for an appropriate interpretation of the analytes.

**Abstract:**

A comprehensive panel of 29 salivary analytes was measured in fattening pigs to evaluate its possible changes along their productive cycle. The identification of those changes would allow a better interpretation of the results according to the productive phase of the animal. Saliva samples were obtained from 49 Large-White pigs (24 females, 25 males) in suckling phase, at the beginning and the end of the nursery phase, and at the beginning and the end of the growing phase. Several analytes changed according to the phase of the productive cycle, with most of the analytes showing higher values at lactation and at the beginning of nursery. Additionally, differences were seen due to sex. When possible relations between performance parameters and analytes were evaluated, significant positive but weak relationships were found between weight at birth and salivary γ-glutamyl transferase, and between back-fat thickness and salivary lactate dehydrogenase. In conclusion, differences in the values of salivary analytes can be found in fattening pigs depending on the productive phase and sex of the animals.

## 1. Introduction

Saliva has gained interest in recent years as a biological sample for analytical purposes [1]. Its collection is easy and non-invasive, does not require the use of specialized material and can be performed by non-trained staff [2,3]. This fact is relevant especially in pigs, since the need for restraint in this species makes blood collection painful and stressful [4,5].

In pigs, saliva has traditionally been used for stress assessment through the measurement of cortisol, a biomarker of the hypothalamic–pituitary–adrenal axis (HPA) activation [4]. More recently, saliva has been also used for the measurement of analytes informing about the health status of the pigs, through additional biomarkers of stress, and biomarkers of inflammation [6], immune response [7], or redox status [8]. In addition to all this, saliva can be used for the measurement of analytes that are routinely determined in blood. This fact has led to the introduction of a term called ‘sialochemistry’, referring to the analytes that can be measured in saliva [9,10]. In veterinary medicine, sialochemistry studies have been previously performed in horses [11], cows [12] and pigs [13]. Namely in the porcine species, a sialochemistry study was made to evaluate the changes in saliva analytes during pregnancy, farrowing and lactation in sows [13].

In pigs, sialochemistry can include analytes providing diverse information. To evaluate the stress response, cortisol, chromogranin A (CgA), salivary α-amylase (sAA), total esterase activity (TEA), butyrylcholinesterase (BChE), lipase (Lip) and oxytocin can be monitored. To assess activation of the immune system, analytes such as the enzyme adenosine deaminase (ADA) and its isoenzymes can be evaluated. Inflammation can be detected by increases in positive acute phase proteins such as haptoglobin (Hp). Redox homeostasis can be assessed by measuring oxidants such as hydrogen peroxide (peroxide-activity Pox-Act, also known as H_2_O_2_), reactive oxygen-derived compounds (d-ROMs) and the advanced oxidation protein products (AOPP), or antioxidants molecules such as cupric reducing antioxidant capacity (CUPRAC), ferric reducing ability of saliva (FRAS), and uric acid (UA). Finally, sialochemistry can include analytes related with the metabolic performance of the organism or with different tissues and organs, which are routinely measured in blood such as total protein (TP), urea, creatinine, glucose, lactate, alanine aminotransferase (ALT), aspartate aminotransferase (AST), alkaline phosphatase (FAL), lactate dehydrogenase (LDH), creatine kinase (CK), γ-glutamil transferase (GGT), calcium (Ca) and phosphorous (P) [14].

The aim of this study was to evaluate whether the aforementioned salivary biomarkers of stress, immunity, inflammation, redox homeostasis and analytes related with metabolism and different tissues and organs that can be included in the sialochemistry profile can change in healthy fattening pigs over their productive cycle and whether they can be influenced by various sources of variations such as gender and performance parameters. For this purpose, a longitudinal study was performed in which a group of animals was serially monitored throughout its productive cycle, from suckling to growing-finishing phase. In addition, the possible influence of other factors or sources of variation in these salivary biomarkers was studied such as: (1) gender of the animals; (2) performance parameters such as weight at birth, weight gain or back fat thickness (BF) in finishing pigs. This data will provide a complete picture of the values of analytes that can be measured in saliva of pigs in farm conditions and their possible physiological influences.

## 2. Materials and Methods

### 2.1. Animals

A total of 50 Large-White pigs (*Sus scrofa domesticus*, 25 males and 25 females) from 5 litters (average litter size of 10.0 ± 0.7) were used in this study which was carried out at the Veterinary Teaching Farm of the University of Murcia (Guadalupe, Murcia, Spain). The birth weight of the piglets was 1.93 ± 0.59 kg (1.83 ± 0.49 for females, 2.02 ± 0.66 for males). This farm is declared free of the porcine respiratory and reproductive syndrome virus, and all animals were vaccinated against *Mycoplasma hyopneumoniae* (Stellamune Mycoplasma, inactivated *Mycoplasma hyopneumoniae* NL 1042, Pfizer Animal Health, Madrid, Spain) and Porcine circovirus type 2 (Porcilis^®^ PCV, MSD Animal Health, Boxmeer, The Netherlands) at weaning.

### 2.2. Experimental Procedure

This experimental was made between October 2021 and April 2022. Animals included in this study were reared under intensive conventional conditions and were monitored throughout their productive cycle, according to the following scheme:

(a) Suckling phase. Cross-fostering was carried out during 24 h postpartum to adjust litter size to 10 piglets per sow. All animals were individually identified by an ear tag. From 10 days of age, suckling piglets had access to a commercial pre-starter diet. Piglets were firstly sampled at 24 days of life (T1), close to weaning, which was performed at 28 days after birth.
(b) Nursery phase. After weaning, piglets were moved to an environmentally controlled nursery, which was in the same farm but in a different building. Each pen contained a standard feeder and a nipple drinker to provide ad libitum access to feed and water. Piglets were fed using a two-phase feeding program over a 7-week period. Pre-starter (for the first 2 weeks, containing 2.54 Mcal/kg Net energy (NE) and 183.2 g/kg crude protein (CP)) and starter diet (for the next 5 weeks, containing 2.52/kg NE and 172.2 g/kg CP). The animals were left a week in the new conditions for acclimatization, after which a new sample was taken (T2). At the end of this phase, the animals were sampled again (T3).
(c) Growing-finishing phase. Pigs were then moved to fattening pens. During this period, animals were given ad libitum access to a nutritionally balanced diet and water. Pig fed an initial growing diet (first 8 weeks, 2.45 Mcal/kg NE, 164 g/kg CP) and a finishing diet (for the next 7 weeks, 2.40 Mcal/kg NE and 149.6 g/kg CP). After one week for acclimatization, pigs were sampled again (T4). The animals stayed in this phase for 14 weeks, being sampled again at the end of the phase (T5).

All diets were based on cereals and soybean meal.

### 2.3. Sampling

Saliva was collected using Salivette tubes (Sarstedt, Aktiengesellschaft and Company, D-51588 Nümbrecht, Germany) containing a sponge (Esponja Marina, La Griega E. Koronis, Madrid, Spain) instead of a cotton swab. Pigs were allowed to chew the sponge, which was clipped to a flexible thin metal rod, for one minute (or more if needed) until thoroughly moist. Then, the sponge was placed into the Salivettes. If an animal was eating or drinking at the sampling time, this animal was sampled later to ensure clean samples (with no evidence of food debris or dirt) were obtained. Tubes were maintained and refrigerated until arrival at the laboratory (<2 h), where the Salivettes were immediately centrifuged (3000× *g*, 10 min, 4 °C), and the supernatants collected. All samples were stored at −80 °C until the end of the experimental sampling period and then analyzed. In order to avoid interferences with the analytical methods, only clean samples were used for analyses.

All samples were taken between 09.30 and 10.30 h. The research protocols were approved by the Bioethical Commission of Murcia University according to the European Council Directives regarding the protection of animals used for experimental purposes (Approval number, 235/2016; Approval date, 25 April 2016).

### 2.4. Welfare Assessment

All animals were monitored in all sampling times for the presence of pathologic conditions, in order to discard any animal suffering lameness, prolapses, gastrointestinal or respiratory diseases, or any other health issue that could interfere with the results. In addition, carcasses were evaluated at the slaughterhouse in order to discard any animals with pathologies detected after sacrifice.

### 2.5. Measurements of Performance Parameters

Table 1 summarizes samplings and measurements throughout the study period. The following data were recorded from the animals:

(a) Weight gain. Pigs were weighed at birth and at each sampling time in order to determine the mean weight gain throughout the study.
(b) Back-fat thickness (BF). It was measured at the end of the growing phase by ultrasound scan using a linear probe (SF1, Wireless Backfat and Loin Depth Scanner, Sonivet, Beijing, China), at the P2 position (last rib, 65 mm from the center line of the back). Measurements were performed twice. The average of the measurements was used for further calculations, following previous protocols [15].

### 2.6. Biomarkers

The following measurements were performed in the saliva samples:

(a) Stress biomarkers. Cortisol was measured by an indirect competitive AlphaLISA assay developed with a commercially available monoclonal antibody against cortisol, a method that was previously validated for porcine saliva [16]. CgA was measured by an in-house method based on previously published protocols [17]. sAA was measured by a commercial spectrophotometric method (a-Amylase, OSR6182, Beckman Coulter Inc., Fullerton, CA, USA) previously validated in porcine saliva [18]. TEA was measured according to a previously validated method [19]. BChE was analyzed using a previously described protocol [20]. Lip was measured by a commercial spectrophotometric method (Lipase, Beckman Coulter Inc., Fullerton, CA, USA). For salivary oxytocin determination, an AlphaLISA method previously developed and validated for use in porcine saliva samples using a monoclonal antibody was used [16].
(b) Inflammatory biomarkers. Hp was measured by an in-house assay based on AlphaLISA technology [13].
(c) Immune system biomarkers. ADA was analyzed with a commercially available spectrophotometric assay (Adenosine Deaminase assay kit, Diazyme Laboratories, Poway, CA, USA), previously validated for porcine saliva [21]. Isoenzymes ADA1 and ADA2 were measured using erythro-9-(2-hydroxy-3-nonyl) adenine (EHNA) as a specific ADA1 inhibitor [7].
(d) Oxidative stress biomarkers. CUPRAC was assayed by the method of [22]. FRAS was measured by the method of [23]. UA was measured using a commercially available kit from Beckman (Beckman Coulter Inc.). AOPP, Pox-Act and d-ROMs concentrations were assessed by previously published methods [24,25,26]. All these assays have been validated in porcine saliva [8].
(e) Routine biochemistry analytes. ALT, AST, ALP, GGT, LDH, CK, urea, creatinine, glucose, lactate, Ca and P were measured using commercial kits from Beckman (Beckman Coulter Inc.). TP was analyzed using a commercial colorimetric kit designed to measure urinary and cerebrospinal fluid (CSF) proteins (Protein in Urine and CSF, Spinreact, Barcelona, Spain).

Assays of the points “c”, “d” and “e” were performed in an automated analyzer (Olympus AU600, Olympus Diagnostica GmbH, Ennis, Ireland). Table S1 shows the different methods with their lower limit of detection.

### 2.7. Statistical Analysis

All data were assessed for normality by the Shapiro–Wilk method. Since most of the data showed non-normal distribution, continuous variables were naturally log transformed prior to analysis. Data showing normal distribution was expressed by mean (standard deviation) whereas data with non-normal distribution was expressed by median (interquartile range). The changes in the different measurements over time were assessed using a Mixed Linear Model in which time and sex were considered as fixed factors, being the individual considered as a random factor. Birth weight, weight gain and BF were used as covariates. Those analytes found as being significantly influenced by covariates were further studied by linear regression analyses in order to know whether the analytical results can be used for prediction of performance parameters. When collinearity was detected, those variables showing the higher significance and no correlation with others were included in the analyses. Statistical analyses were performed using the SPSS statistics package (IBM SPSS Statistics for Windows, Version 26.0. IBM Corp., Armonk, NY, USA), and the significance was set at *p* < 0.05.

## 3. Results

### 3.1. Longitudinal Study

From the 50 pigs selected initially, 49 finished the study with no pathologic conditions, therefore they were included in the study, whereas one pig was discarded due to lameness. Data regarding the animals included in the longitudinal study are shown in Table 2.

The results of the salivary biomarkers at each sampling period appear in Table 3. Almost all the analytes showed significant changes in their values along the different sampling times. According to the results, biomarkers can be divided in those which concentration increases or decrease throughout productive cycle of the animals.

Most of the analytes showed a significant decrease in their values along time. The analytes that decreased can be classified into two groups: (1) analytes that started to decrease their concentration at the end of the growing phase (sAA, TEA, BChE, oxytocin, Hp, FRAS, ALT, ALP, GGT, LDH, CK, Ca and TP); and (2) analytes that started to decrease their concentration at nursery. In this second group, two sub-groups could be differentiated: (a) analytes that increased later at growing phase, but did not reach the values at lactation (ADA1, ADA2, AOPP and AST); and (b) analytes that returned to initial values at the end of the growing phase (CgA, Lip, CUPRAC, UA and urea).

On the other hand, some analytes showed significant increases with time in their concentration. These analytes can be also sub-divided into three groups: (1) those that increased and remained high at the end of the growing phase (creatinine); (2) analytes that increased at the nursery period, then returned to similar values than at lactation at the end of the growing phase (d-ROMs and glucose); and (3) analytes that increased at nursery followed by a decrease under lactation values by the end of the growing phase (cortisol, lactate and P).

Finally, Pox-Act concentrations did not show significant changes between sampling times.

Different values between sexes were observed in some analytes. Females showed higher values of TEA, Lip, CUPRAC, FRAS, UA, AOPP, ALT, AST, GGT, CK, urea, glucose, P and TP. Those differences were mainly seen by the end of nursery and growing phases.

### 3.2. Performance Data Influences on the Analytes

Table 4 shows the linear regression analyses results performed with those covariates that significantly affected the salivary biomarkers. Only a small percentage of the variability of the dependent variables could be predicted by salivary biomarkers.

## 4. Discussion

In this report, a comprehensive panel of 29 analytes was measured in saliva from fattening pigs that were serially monitored throughout their productive cycle. These analytes have been previously described in other studies [13,14], but the evolution of this profile of analytes during fattening has not been evaluated. The knowledge of how biomarkers could change during the different phases of fattening would be of interest for an adequate interpretation of the results and will contribute to a wider use of these analytes for the evaluation of pig health and welfare in farm conditions. The knowledge of the values of biomarkers in saliva in a healthy state can be considered as a basis for an appropriate use of them for diagnosis and prevention.

For the biomarkers’ measurement, several analytical techniques were used. Most of the analytes measured in this report were present in saliva in sufficient quantity to be quantified by spectrophotometric assays. This has some advantages since the reagents for these methods are usually not expensive and also these assays can be easily set up at the laboratory. In addition, they can be automated, allowing the measurement of large panels of analytes in a short time and with a small volume of sample. In this report, a total of 25 analytes were measured by spectrophotometric automated methods. On the other hand, other biomarkers that are present in a very low concentration require more sensitive techniques. This is the case of cortisol, CgA, Hp and oxytocin that were quantified by fluorometric methods or AlphaLISA technology, which are highly sensitive and allow the detection of analytes present in a very small concentration [16]. The volume obtained at the different sampling times was enough for the measurement of all the panels of analytes. However, growing pigs provided a high volume of saliva samples, whereas piglets at lactation were less prone to chew the sponges, and generally more time was required to obtain the sufficient volume of samples.

Almost all analytes measured in this trial changed according to the productive phase of the pigs. The variations found throughout the productive stage were different depending on the analytes, but in general most of the analytes decreased their concentration at the nursery and growing phases if compared with lactation. Some of them decreased at the beginning of the nursery phase, such as CgA, TEA, BChE, Lip, oxytocin, ADA, ALT, AST, GGT, CK, urea, Ca and TP, whereas others decreased by the end of nursery or at growing phases, such as sAA, Hp, ALP and redox biomarkers such as CUPRAC, FRAS, UA and AOPP. Among the possible factors that could be involved in these variations, it could be postulated the growing of the animals as well as the dietetic changes among the different phases. Aging can affect salivary components, as it has been previously demonstrated in human saliva [27,28].

The values of the different analytes obtained in this manuscript were in line with those previously reported for healthy animals in the growing phase. The range of values previously reported were: 73.6–320.0 IU/L for TEA; 0.35–0.69.4 IU/mL for sAA; 0.17–2.84 UI/mL for BChE; 17.0–598.3 IU/L for Lip; 0.25–12.00 ng/mL for oxytocin; 0.12–1.56 UI/mL for ADA1; 0.68–15.26 IU/L for ADA2; 24.5–536.0 µmol/L for CUPRAC; 32.0–885.0 µmol/L for FRAS; 0.02–1.05 mg/dL for UA; and 23.4–378.4 µmol/L for AOPP [16,29,30]. This could be considered as values for healthy growing pigs since pigs of our study did not have evidence of any disease during the experimental period. In addition, most of those analytes showed higher values in animals suffering pain or discomfort due to pathologic conditions such as lameness or rectal prolapse [29], and in a previous report, LDH showed values between 0.1–0.7 IU/mL, also similar to the values indicated in this research, and increases in this biomarker were detected in lame pigs and after applying a stress by snaring [31]. CgA values previously reported in healthy growing pigs were between 0.40–1.20 µg/mL, also being similar to the values obtained in this manuscript for animals in the same productive phase [32]. In general, and also in agreement with other reports, all analytes showed a high inter-individual variability based on the high SD and IR obtained. This fact means that if saliva is going to be used as a specimen for biomarker analysis, ideally each individual should act as its own control in order to avoid the individual differences.

In our experimental conditions, there were analytes that markedly increased at the beginning of the nursery phase, such as cortisol, glucose and lactate. Weaning is highly stressful for the animals, and increases in salivary stress biomarkers have been found in the very close period to weaning [33]. Although animals were left one week for acclimation, the 1.8-fold increase in cortisol observed in our trial at the beginning of the nursery period with respect to lactation levels could indicate that stress was still present in those animals. This increase was similar to the 1.5-fold increase previously reported one day after weaning [33]. Lactate also increase due to stress [34,35] as well as glucose [36]. In addition, other reasons such as the change in pancreatic function that occurs when a change in the diet from liquid to solid occurs could influence on the values of glucose [37]. In any case, the increases found in these analytes confirm that a special care should be taken in weaning in order to try to minimize the stress that is produced in this situation.

Sex of the animals also can influence results in some analytes. Enzymes such as TEA, Lip, ALT, AST, GGT, and CK, redox biomarkers such as CUPRAC, FRAS, UA and AOPP, and metabolites such as urea, glucose, P and TP were higher in females than in males in selected sampling days. Sex-related differences in the salivary proteome have been identified in humans, with 65 proteins being differently expressed between males and females [38]. Additionally, sex differences have been also found in pigs in levels of some analytes such as acute phase proteins [39,40]. Further studies should be performed to elucidate the reasons for these differences, but in any case, this raises the importance of take into consideration the sex when interpreting certain analytes in saliva. In addition, in the case of female pigs, the productive stage the animal is in should be considered. For example, salivary cortisol, BChE, Lip, ADA, Hp, AOPP and lactate have been reported to change in sows depending on the time of the gestation, showing in general a significant increase at peripartum with the exception of salivary Lip [13].

Linear regression analyses detected some relationship between selected performance parameters and some biomarkers. However, only a few percentages of the changes in performance parameters can be explained by the changes recorded in the salivary biomarkers, maybe due to the reason that the recorded weights and BF were highly homogeneous among animals. Possibly, the inclusion of other models comparing pigs with different productive performances would yield more analytes implied. In addition, only healthy animals with a standard growth and development were included in this trial, since the objective of this report was to study changes in healthy individuals. Therefore, results were not compared with animals with poor performance parameters, pathologic conditions or under inadequate welfare conditions. This could have affected the results obtained in the regression assays, leading to the low predictive value observed for the different biomarkers. In our conditions, GGT showed a positive relationship with the birth weight of the piglets. In cows, a relationship has been found between GGT levels in serum and immunoglobulin G transference in colostrum [41], so it could be postulated that a higher GGT at birth could indicate a better immune status of the animal that could be related with birth weight. LDH had a weak negative relationship with BF at the end of the growing phase. Since LDH is present in muscle, it could be postulated higher level of this enzyme could related with higher lean mass of the animal.

## 5. Conclusions

When a comprehensive panel of 29 analytes is measured in saliva from the same healthy fattening pigs in farm conditions during their productive cycle, differences can be found depending on the productive phase and sex. These differences can be related in some cases with performance parameters and should be taken into consideration for an appropriate interpretation of the analytes. Nevertheless, further studies should be performed including animals with compromised welfare and/or health conditions in order to be compared with healthy animals of the same sex and productive stage, in order to evaluate the potential of these analytes to act as biomarkers of these conditions.

## Figures and Tables

**Table 1:** Summary of the experimental protocol.

Sampling Number	Phase	Approximate Age of the Animals (Days)	Measurements/Sampling
T0	Birth time	1–2	Body weight
T1	Suckling phase	24	Body weight Saliva
T2	Beginning of nursery phase	35	Body weight Saliva
T3	End of the nursery phase	88	Body weight Saliva
T4	Beginning of the growing phase	99	Body weight Saliva
T5	End of the growing phase	193	Body weight BF Saliva

BF: back-fat thickness.

**Table 2:** Data from the animals included in the longitudinal study. Mean (standard deviation) are expressed in bold, whereas median (interquartile range) are expressed in italics.

Number of animals	49
Females	24
Males	25
Weight at birth (kg)	*1.84 (0.83)*
Females	*1.72 (0.72)*
Males	*2.05 (1.03)*
Weight gain (gr/day)	**638.20** (**79.72**)
Females	**596.91** (**69.06**)
Males	676.04 *** (**70.40**)
BF (cm)	**12.61** (**3.01**)
Females	**11.92** (**3.23**)
Males	13.22 ** (**2.73**)

BF: Back-fat thickness. Asterisks show statistically significant differences between sexes (**: *p* < 0.01; ***: *p* < 0.001).

**Table 3:** Results obtained in a panel of 29 salivary biomarkers in 49 Large-White pigs (24 females, 25 males) at lactation (T1), beginning of the nursery (T2), end of nursery (T3), beginning of growing (T4) and end of growing (T5). Mean (standard deviation) is expressed in bold, whereas median (interquartile range) is expressed in italics.

	Sampling Time							
Biomarkers	T1	T2	T3	T4	T5	Fixed Factors	*p* Value	Significant Covariates
Cortisol (ng/mL)	*125.6* ª *(102.2)*	*224.4* ^b^ *(233.4)*	*60.8* ^c^ *(32.6)*	*83.6* ^c^ *(53.2)*	*55.6* ^c^ *(58.0)*	Time	<0.001	
Female	*119.0* ^a^ *(108.4)*	*253.4* ^b^ *(249.4)*	*66.0* ^c^ *(36.3)*	*88.4* ^c^ *(49.2)*	*74.6* ^c^ *(51.2)*	Sex	0.208	
Male	*129.2* ^a^ *(104.2)*	*214.4* ^b^ *(150.8)*	*58.4* ^c^ *(32.6)*	*78.4* ^ac^ *(53.0)*	*49.2* ^c^ *(51.8)*	Time × sex	0.855	
CgA (µg/mL)	**0.61**^a^ (**0.26**)	*0.39* ^b^ *(0.33)*	*0.36* ^b^ *(0.42)*	*0.49* ^ab^ *(0.39)*	**0.64**^a^ (**0.32**)	Time	<0.001	BF (*p* = 0.004)
Female	**0.60**^ab^ (**0.26**)	*0.37* ^b^ *(0.39)*	*0.44* ^ab^ *(0.42)*	*0.49* ^ab^ *(0.57)*	**0.69**^a^ (**0.35**)	Sex	0.893	
Male	**0.62**^a^ (**0.27**)	*0.41* ^ab^ *(0.30)*	*0.24* ^b^ *(0.96)*	0.46 ^ab^ (0.42)	**0.60**^a^ (**0.29**)	Time × sex	0.726	
sAA (IU/mL)	*9.22* ^a^ *(35.26)*	*9.22* ^b^ *(10.57)*	*2.12* ^c^ *(4.12)*	*0.63* ^d^ *(1.24)*	*0.56* ^d^ *(0.86)*	Time	<0.001	
Female	*9.22* ^a^ *(41.50)*	*9.22* ^a^ *(10.95)*	*2.41* ^b^ *(5.75)*	*0.66* ^c^ *(1.61)*	*0.80* ^c^ *(1.21)*	Sex	0.539	
Male	*9.22* ^a^ *(20.43)*	*8.82* ^a^ *(11.49)*	*1.89* ^b^ *(3.41)*	*0.48* ^c^ *(1.01)*	*0.44* ^c^ *(0.58)*	Time × sex	0.756	
TEA (IU/L)	**769.5**^a^ (**401.8**)	**320.8**^b^ (**146.6**)	**179.5**^cd^ (**78.9**)	**136.4**^d^ (**68.7**)	*168.3* ^c^ *(146.6)*	Time	<0.001	
Female	*840.0* ^a^ *(651.3)*	*326.9* ^b^ *(143.7)*	**203.8**^c^ (**63.1**)	*117.9* ^d^ *(66.6)*	*227.5* ^bc^ *(158.9)*	Sex	0.028	
Male	*559.9* *^a^ *(292.9)*	*254.5* ^b^ *(150.8)*	156.3^*c^ (**86.5**)	*146.1* ^c^ *(96.5)*	*135.1* *^c^ *(109.8)*	Time × sex	0.035	
BChE (IU/mL)	*1.49* ^a^ *(1.33)*	*0.88* ^b^ *(0.65)*	*0.69* ^bc^ *(0.78)*	*0.40* ^c^ *(0.71)*	*0.59* ^b^ *(1.32)*	Time	<0.001	BF (*p* = 0.028)
Female	*1.63* ^a^ *(1.24)*	*0.94* ^a^ *(0.47)*	*0.80* ^ab^ *(1.21)*	*0.40* ^b^ *(0.58)*	*0.64* ^a^ *(2.12)*	Sex	0.491	
Male	*1.37* ^a^ *(1.60)*	*0.72* ^b^ *(0.85)*	*0.55* ^b^ *(0.60)*	*0.39* ^b^ *(1.23)*	*0.36* ^b^ *(0.92)*	Time × sex	0.351	
Lip (IU/L)	*44.0* ^a^ *(70.4)*	*16.6* ^c^ *(37.0)*	*30.4* ^b^ *(29.6)*	*44.0* ^ab^ *(70.6)*	*32.4* ^ab^ *(47.2)*	Time	<0.001	
Female	*44.4* ^a^ *(43.2)*	*18.8* ^c^ *(42.4)*	*41.6* ^abc^ *(32.7)*	*42.0* ^abc^ *(90.7)*	*48.2* ^ab^ *(85.0)*	Sex	0.030	
Male	*42.0* ^a^ *(83.6)*	*14.4* ^c^ *(24.6)*	*25.6* *^bc^ *(24.8)*	*46.4* ^a^ *(65.6)*	*24.0* *^ab^ *(28.2)*	Time × sex	0.384	
Oxytocin (ng/mL)	*6.16* ^a^ *(7.17)*	*3.62* ^a^ *(3.06)*	*1.33* ^b^ *(0.72)*	*1.64* ^b^ *(0.91)*	*1.74* ^b^ *(0.77)*	Time	<0.001	
Female	*6.71* ^a^ *(6.98)*	*4.48* ^a^ *(5.06)*	*1.50* ^b^ *(0.93)*	*1.57* ^b^ *(1.13)*	*2.07* ^b^ *(1.60)*	Sex	0.201	
Male	*4.85* ^a^ *(7.53)*	*3.24* ^a^ *(2.60)*	*1.24* ^b^ *(0.58)*	*1.67* ^b^ *(0.95)*	*1.53* ^b^ *(0.52)*	Time × sex	0.077	
Hp (µg/mL)	*3.36* ^a^ *(2.79)*	*3.26* ^a^ *(1.90)*	*1.07* ^b^ *(1.17)*	*0.67* ^b^ *(0.99)*	*0.54* ^b^ *(0.84)*	Time	<0.001	
Female	*3.44* ^a^ *(3.26)*	*3.59* ^a^ *(2.06)*	*1.48* ^b^ *(1.28)*	*0.60* ^b^ *(0.96)*	*0.85* ^b^ *(0.93)*	Sex	0.276	
Male	*3.36* ^a^ *(2.33)*	*3.27* ^a^ *(1.82)*	*0.75* *^b^ *(0.64)*	*0.72* ^b^ *(1.03)*	*0.44* ^b^ *(0.40)*	Time × sex	0.111	
ADA1 (IU/mL)	*3.90* ^a^ *(3.15)*	*2.71* ^ab^ *(1.37)*	*2.03* ^c^ *(1.78)*	*1.14* ^d^ *(0.97)*	*2.13* ^bc^ *(1.62)*	Time	<0.001	
Female	*3.90* ^a^ *(5.60)*	*2.71* ^ab^ *(2.04)*	*2.51* ^b^ *(1.18)*	*1.17* ^c^ *(0.84)*	*2.38* ^b^ *(1.25)*	Sex	0.162	
Male	*3.87* ^a^ *(2.63)*	*2.71* ^ab^ *(1.02)*	*1.54* ^c^ *(2.01)*	*1.09* ^c^ *(1.12)*	*1.69* ^bc^ *(1.57)*	Time × sex	0.409	
ADA2 (IU/L)	*11.84* ^a^ *(8.95)*	*7.38* ^b^ *(4.67)*	*4.77* ^cd^ *(6.27)*	*3.70* ^d^ *(3.37)*	*5.53* ^c^ *(5.80)*	Time	<0.001	
Female	*12.20* ^a^ *(6.54)*	*7.54* ^ab^ *(4.37)*	*5.91* ^bc^ *(5.01)*	*3.68* ^c^ *(4.19)*	*6.51* ^ab^ *(4.39)*	Sex	0.636	
Male	*11.24* ^a^ *(12.06)*	*7.22* ^a^ *(4.68)*	*3.12* ^b^ *(5.10)*	*3.72* ^b^ *(3.39)*	*4.40* ^b^ *(5.36)*	Time × sex	0.092	
CUPRAC (µmol/L)	**287.9**^a^ (**114.3**)	*279.2* ^a^ *(205.7)*	*146.4* ^b^ *(128.0)*	*222.0* ^ab^ *(236.9)*	*195.6* ^ab^ *(205.6)*	Time	<0.001	Birth weight (*p* = 0.045)
Female	**299.2**^a^ (**131.6**)	*293.6* ^a^ *(182.0)*	*217.0* ^a^ *(176.8)*	*218.0* ^a^ *(260.8)*	*264.8* ^a^ *(190.9)*	Sex	0.004	
Male	**277.5**^a^ (**97.4**)	*264.0* ^a^ *(231.2)*	*130.8* ***^b^ *(94.4)*	*222.8* ^a^ *(230.8)*	*156.0* **^ab^ *(127.6)*	Time × sex	0.183	
FRAS (µmol/L)	*445.2* ^a^ *(391.2)*	*378.0* ^ab^ *(325.2)*	*193.2* ^c^ *(189.6)*	*239.2* ^bc^ *(306.6)*	*243.2* ^c^ *(243.6)*	Time	<0.001	Weight gain (*p* = 0.048)
Female	*539.8* ^a^ *(538.4)*	*398.8* ^ab^ *(453.6)*	*273.2* ^b^ *(262.1)*	*192.8* ^b^ *(335.2)*	*368.8* ^b^ *(286.3)*	Sex	<0.001	
Male	*426.0* *^a^ *(190.8)*	*334.2* ^a^ *(254.2)*	*143.6* ***^c^ *(155.0)*	*256.0* ^ab^ *(301.8)*	*202.8* **^bc^ *(136.8)*	Time × sex	0.144	
UA (mg/dL)	*0.52* ^a^ *(0.36)*	*0.40* ^a^ *(0.31)*	*0.20* ^b^ *(0.18)*	*0.16* ^b^ *(0.18)*	*0.44* ^a^ *(0.32)*	Time	<0.001	Birth weight (*p* = 0.004) Weight gain (*p* = 0.041)
Female	*0.68* ^a^ *(0.84)*	*0.40* ^b^ *(0.36)*	*0.24* ^bc^ *(0.23)*	*0.16* ^c^ *(0.19)*	*0.46* ^ab^ *(0.30)*	Sex	0.011	
Male	*0.44* ***^a^ *(0.28)*	*0.40* ^a^ *(0.30)*	*0.16* *^b^ *(0.12)*	*0.20* ^b^ *(0.16)*	*0.40* ^a^ *(0.30)*	Time × sex	0.049	
AOPP (µmol/L)	*214.9* ^a^ *(278.9)*	*214.4* ^a^ *(221.0)*	*69.0* ^b^ *(92.8)*	*92.8* ^b^ *(196.9)*	*102.2* ^b^ *(165.0)*	Time	<0.001	Weight gain (*p* = 0.013)
Female	*244.0* ^a^ *(336.8)*	*201.0* ^a^ *(237.4)*	*97.7* ^b^ *(108.4)*	*84.0* ^b^ *(191.8)*	*167.0* ^ab^ *(144.5)*	Sex	0.006	
Male	*207.9* ^a^ *(171.2)*	*215.7* ^a^ *(187.0)*	*30.6* **^c^ *(66.4)*	*132.6* ^ab^ *(222.3)*	*63.8* *^bc^ *(91.7)*	Time×sex	0.019	
Pox-Act (µmol/L)	*501.6* ^a^ *(532.4)*	*309.6* ^a^ *(271.9)*	*331.2* ^a^ *(413.1)*	*528.7* ^a^ *(781.0)*	*360.6* ^a^ *(346.2)*	Time	0.018	Weight gain (*p* = 0.017)
Female	*454.3* ^a^ *(473.6)*	*251.0* ^a^ *(196.1)*	*437.6* ^a^ *(465.3)*	*422.4* ^a^ *(577.6)*	*384.1* ^a^ *(496.7)*	Sex	0.111	
Male	*583.8* ^ab^ *(617.1)*	*330.0* ^ab^ *(347.0)*	*307.0* ^a^ *(409.9)*	*683.2* *^b^ *(1116.8)*	*346.4* ^a^ *(243.5)*	Time × sex	0.021	
d-Roms (Carrateli units)	*206.4* ^a^ *(121.8)*	*203.2* ^ab^ *(134.7)*	**298.9**^bc^ (**136.2**)	*287.0* ^c^ *(282.2)*	*218.8* ^ab^ *(55.2)*	Time	<0.001	
Female	*240.0* ^a^ *(128.4)*	*219.2* ^a^ *(112.4)*	*334.2* ^a^ *(195.2)*	*196.4* ^a^ *(311.6)*	*218.2* ^a^ *(101.9)*	Sex	0.962	
Male	*188.4* ^a^ *(86.8)*	*201.6* ^a^ *(204.4)*	*227.6* ^a^ *(166.8)*	*325.6* ^b^ *(186.6)*	*219.6* ^a^ *(27.2)*	Time × sex	0.016	
ALT (IU/L)	*42.2* ^a^ *(81.7)*	*18.2* ^b^ *(25.2)*	*6.4* ^d^ *(9.6)*	*8.4* ^cd^ *(19.6)*	*14.4* ^bc^ *(22.4)*	Time	<0.001	Weight gain (*p* = 0.022)
Female	*59.6* ^a^ *(80.4)*	*19.6* ^b^ *(26.0)*	*10.8* ^b^ *(19.0)*	*9.6* ^b^ *(21.0)*	*18.0* ^b^ *(20.5)*	Sex	0.032	
Male	*34.4* ^a^ *(87.0)*	*17.2* ^ab^ *(25.0)*	*4.8* ***^c^ *(5.6)*	*8.0* ^b^ *(19.2)*	*14.4* ^b^ *(26.2)*	Time × sex	0.020	
AST (IU/L)	*454.4* ^a^ *(441.6)*	*101.0* ^b^ *(81.2)*	*61.2* ^d^ *(61.6)*	*67.2* ^c^ *(68.2)*	*73.2* ^bc^ *(96.0)*	Time	<0.001	Birth weight (*p* = 0.047)
Female	**659.7**^a^ (**313.2**)	*107.2* ^b^ *(90.8)*	*72.4* ^b^ *(62.8)*	*70.4* ^b^ *(44.6)*	*101.2* ^b^ *(87.2)*	Sex	0.020	
Male	**411.6** *^a^ (**182.2**)	*91.6* ^b^ *(91.0)*	*41.2* *^d^ *(45.6)*	*66.8* ^bcd^ *(79.4)*	*60.8* ^bc^ *(78.6)*	Time × sex	0.426	
ALP (IU/L)	*179.2* ^a^ *(214.4)*	*82.4* ^ab^ *(91.6)*	*72.8* ^a^ *(78.0)*	*65.6* ^a^ *(71.2)*	*31.2* ^b^ *(40.8)*	Time	0.002	
Female	*187.2* ^ab^ *(264.0)*	*83.2* ^ab^ *(110.4)*	*91.6* ^a^ *(101.0)*	*49.6* ^ab^ *(106.2)*	*45.2* ^b^ *(60.0)*	Sex	0.354	
Male	*144.0* ^a^ *(162.2)*	*72.8* ^ab^ *(95.6)*	*41.6* **^ab^ *(52.0)*	*75.2* ^a^ *(72.4)*	*29.6* ^b^ *(18.0)*	Time × sex	0.003	
GGT (IU/L)	*27.8* ^a^ *(17.5)*	*12.7* ^b^ *(12.8)*	*9.8* ^b^ *(12.0)*	*9.4* ^b^ *(9.4)*	*9.1* ^b^ *(8.1)*	Time	<0.001	Birth weight (*p* = 0.015)
Female	*28.8* ^a^ *(31.6)*	*13.4* ^b^ *(14.5)*	*12.6* ^b^ *(16.6)*	*8.1* ^b^ *(7.4)*	*11.9* ^b^ *(11.2)*	Sex	0.037	
Male	*26.8* ^a^ *(11.2)*	*11.9* ^b^ *(14.0)*	*7.2* **^b^ *(7.3)*	*10.3* ^b^ *(11.4)*	*7.1* **^b^ *(4.3)*	Time × sex	0.010	
LDH (IU/mL)	*2.38* ^a^ *(1.90)*	*0.50* ^b^ *(0.37)*	*0.32* ^c^ *(0.37)*	*0.23* ^c^ *(0.21)*	*0.22* ^c^ *(0.30)*	Time	<0.001	BF (*p* = 0.027)
Female	*2.71* ^a^ *(3.16)*	*0.52* ^b^ *(0.36)*	*0.38* ^bc^ *(0.37)*	*0.23* ^c^ *(0.19)*	*0.35* ^bc^ *(0.33)*	Sex	0.149	
Male	*2.23* ^a^ *(1.41)*	*0.39* ^b^ *(0.36)*	*0.20* ^c^ *(0.29)*	*0.21* ^bc^ *(0.27)*	*0.21* ^bc^ *(0.23)*	Time × sex	0.701	
CK (IU/L)	*49.4* ^a^ *(66.7)*	*11.6* ^b^ *(8.9)*	*4.6* ^d^ *(6.0)*	*6.0* ^cd^ *(6.2)*	*7.8* ^bc^ *(8.0)*	Time	<0.001	
Female	*76.4* ^a^ *(113.0)*	*12.2* ^b^ *(8.0)*	*6.1* ^c^ *(6.7)*	*6.1* ^c^ *(5.3)*	*10.1* ^bc^ *(9.5)*	Sex	0.003	
Male	*42.8* ***^a^ *(34.0)*	*9.6* ^b^ *(10.2)*	*3.7* *^d^ *(3.5)*	*5.3* ^cd^ *(6.7)*	*6.8* *^bc^ *(5.2)*	Time × sex	0.115	
Urea (mg/dL)	**14.4**^ac^ (**7.6**)	*0.4* ^b^ *(5.8)*	*3.2* ^b^ *(8.0)*	*11.6* ^a^ *(8.6)*	*21.6* ^c^ *(14.8)*	Time	<0.001	
Female	*14.0* ^a^ *(10.8)*	*1.2* ^b^ *(11.6)*	*5.4* ^a^ *(11.1)*	*12.2* ^a^ *(11.0)*	*24.6* ^c^ *(11.4)*	Sex	0.050	
Male	*13.2* ^a^ *(13.0)*	*0.1* ^b^ *(4.8)*	*1.2* ***^b^ *(5.4)*	*10.4* ^a^ *(8.0)*	*16.4* *^a^ *(12.6)*	Time×sex	0.015	
Creatinine (mg/dL)	*0.66* ^a^ *(0.60)*	*1.02* ^b^ *(0.97)*	*0.68* ^a^ *(0.48)*	*0.60* ^a^ *(0.64)*	*1.52* ^c^ *(1.00)*	Time	<0.001	
Female	*0.48* ^a^ *(0.48)*	*1.08* ^b^ *(0.92)*	*0.80* ^ab^ *(0.61)*	*0.52* ^a^ *(0.72)*	*1.96* ^c^ *(1.16)*	Sex	0.082	
Male	*0.68* ^a^ *(0.66)*	*0.92* ^b^ *(1.10)*	0.44 **^a^ (0.34)	*0.76* ^a^ *(0.64)*	*1.44* **^b^ *(0.96)*	Time × sex	0.004	
Glucose (mg/dL)	*0.8* ^a^ *(3.8)*	*87.5* ^b^ *(101.8)*	*2.3* ^a^ *(2.6)*	*2.8* ^a^ *(4.6)*	*1.6* ^a^ *(1.8)*	Time	<0.001	
Female	*0.9* ^a^ *(3.6)*	*93.7* ^b^ *(101.4)*	*2.9* ^a^ *(6.1)*	*3.5* ^a^ *(4.3)*	*2.4* ^a^ *(2.2)*	Sex	0.049	
Male	*0.8* ^a^ *(3.9)*	*66.1* ^b^ *(99.8)*	*1.8* *^a^ *(2.4)*	*1.8* ^a^ *(5.5)*	1.2 *^a^ (1.1)	Time × sex	0.349	
Lactate (µmol/L)	*145.5* ^a^ *(384.6)*	*2819.1* ^b^ *(2688.2)*	*58.2* ^c^ *(110.2)*	*49.9* ^cd^ *(78.8)*	*16.6* ^d^ *(41.6)*	Time	<0.001	Weight gain (*p* = 0.012)
Female	*183.0* ^a^ *(324.3)*	*2752.6* ^b^ *(1405.4)*	*54.1* ^a^ *(237.0)*	*54.1* ^a^ *(94.1)*	*16.6* ^a^ *(49.9)*	Sex	0.776	
Male	*116.4* ^a^ *(602.9)*	*3027.0* ^b^ *(3937.6)*	*58.2* ^a^ *(68.6)*	*41.6* ^a^ *(57.2)*	*16.6* ^a^ *(37.4)*	Time × sex	0.264	
Ca (mg/dL)	**13.4**^a^ (**2.9**)	**8.9**^b^ (**2.1**)	**5.9**^d^ (**2.4**)	**7.3**^cd^ (**2.5**)	**7.7**^bc^ (**2.0**)	Time	<0.001	Weight gain (*p* = 0.040)
Female	**14.1**^a^ (**2.9**)	*8.5* ^b^ *(2.4)*	**5.9**^c^ (**2.3**)	**7.4**^bc^ (**2.4**)	**8.4**^b^ (**1.7**)	Sex	0.158	
Male	**12.7**^a^ (**2.9**)	*9.1* ^b^ *(2.2)*	**5.8**^c^ (**2.5**)	**7.3**^bc^ (**2.5**)	**7.1**^c^ (**2.1**)	Time × sex	0.014	
P (mg/dL)	**1.57**^a^ (**0.54**)	*1.96* ^b^ *(2.37)*	*1.28* ^ac^ *(0.54)*	*1.44* ^ab^ *(1.46)*	*0.96* ^c^ *(0.88)*	Time	<0.001	Birth weight (*p* = 0.030) Weight gain (*p* = 0.026)
Female	**1.74**^ab^ (**0.64**)	*2.00* ^b^ *(2.08)*	*1.40* ^ab^ *(0.80)*	*1.46* ^ab^ *(1.37)*	*1.20* ^a^ *(0.74)*	Sex	0.006	
Male	**1.41** *^abc^ (**0.38**)	*1.92* ^b^ *(2.54)*	*1.20* **^ac^ *(0.44)*	*1.44* ^ab^ *(1.54)*	*0.84* *^c^ *(0.56)*	Time × sex	0.875	
Protein (mg/dL)	*297.2* ^a^ *(323.9)*	*170.3* ^b^ *(152.3)*	*44.1* ^d^ *(75.8)*	*62.8* ^cd^ *(61.9)*	**85.0**^c^ (**37.9**)	Time	<0.001	Weight gain (*p* = 0.026) BF (*p* = 0.020)
Female	*446.2* ^a^ *(400.0)*	*201.8* ^b^ *(191.5)*	*77.2* ^c^ *(76.0)*	*54.5* ^c^ *(97.1)*	*76.4* ^c^ *(54.9)*	Sex	0.014	
Male	*278.2* ^a^ *(171.7)*	*166.9* ^b^ *(169.4)*	*26.9* ***^d^ *(35.8)*	*65.9* ^c^ *(40.2)*	*79.0* ^bc^ *(48.2)*	Time × sex	0.018	

CgA: chromogranin A; sAA: salivary α-amylase; TEA: total esterase activity; BChE: butyrylcholinesterase; Lip: lipase; Hp: haptoglobin; ADA1 and 2: adenosine deaminase isoenzymes 1 and 2; CUPRAC: cupric reducing antioxidant capacity; FRAS: ferric reducing ability of saliva; UA: uric acid; AOPP: advanced oxidation protein products; Pox-Act: hydrogen peroxide; d-ROMs: reactive oxygen-derived compounds (d-ROMs); AST: aspartate aminotransferase; ALT: alanine aminotransferase; ALP: alkaline phosphatase; GGT: γ-glutamil transferase; LDH: lactate dehydrogenase; CK: creatine kinase; Ca: calcium; P: phosphorous. Statistical analysis: a different letter indicates significant differences between sampling times; asterisks indicate significant differences between sexes (*: *p* < 0.05; **: *p* < 0.01; ***: *p* < 0.001).

**Table 4:** Linear regression analysis results obtained between some performance parameters and analytical variables measured in saliva.

Dependent Variables	Model R^2^	Overall *p* Value	Constant	Predictors Included in the Model	B Coefficient	Predictors *p* Value
Birth weight	0.07	<0.001	1.253	GGT	−0.08	<0.001
Weight gain	None					
BF	0.04	0.002	2.846	LDH	−0.04	0.002

BF: Back fat thickness; GGT: γ-glutamil transferase; LDH: lactate dehydrogenase.

## Data Availability

The data presented in this study are available on request from the corresponding author.

## Supplementary Materials

The following supporting information can be downloaded at: https://www.mdpi.com/article/10.3390/ani12141865/s1, Table S1: Analytical methods employed and their lower limits of detection (LLOD).
