# Supplementary material for: Changes in a Comprehensive Profile of Saliva Analytes in Fattening Pigs during a Complete Productive Cycle: A Longitudinal Study
Source: Animals (Basel). 2022 Jul 21;12(14):1865. doi: 10.3390/ani12141865 (PMC9312009; doi:10.3390/ani12141865)
Supplement: Supplementary file 1 [file animals-12-01865-s001.zip › animals-1824154 - Supplementary.pdf]

animals

MDPI

Table S1. Analytical methods employed and their lower limits of detection (LLOD).

Method (Units)	Reagent Provider	LLOD
Cortisol (ng/mL)	AlphaLISA based in-house method	0.22
CgA (µg/mL)	TR-IFMA based in house method	24.53×10^{-3}
sAA (IU/mL)	Beckman [a]	2.0×10^{-3}
TEA (IU/L)	Colorimetric in-house method	12.24
BChE (IU/mL)	Colorimetric in-house method	2.5×10^{-3}
Lip (IU/L)	Beckman	1.0
Oxytocin (pg/mL)	AlphaLISA based in-house method	115.6
Hp (µg/mL)	AlphaLISA based in-house method	22.5×10^{-3}
ADA1 (IU/mL)	Dyazime [b]	0.07×10^{-3}
ADA 2 (IU/L)	Dyazime	0.07
CUPRAC (µmol/L)	Colorimetric in-house method	0.0286×10^{-3}
FRAS (µmol/L)	Colorimetric in-house method	0.0146×10^{-3}
UA (mg/dL)	Beckman	0.08
AOPP (µmol/L)	Colorimetric in-house method	3.83
Pox-Act (µmol/L)	Colorimetric in-house method	5.28
d-ROMs (Carr. Units)	Colorimetric in-house method	8.57
ALT (IU/L)	Beckman	1.0
AST (IU/L)	Beckman	1.0
ALP (IU/L)	Beckman	1.0
GGT (IU/L)	Beckman	1.0
LDH (IU/L)	Beckman	3.0
CK (IU/L)	Beckman	3.0
Urea (mg/dL)	Beckman	0.78
Creatinine (mg/dL)	Beckman	0.04
Glucose (mg/dL)	Beckman	0.73
Lactate (mmol/L)	Beckman	0.01
Calcium (mg/dL)	Beckman	0.12
Phosphorous (mg/dL)	Beckman	0.31
Proteins (mg/dL)	Spinreact [c]	0.1

TR-IFMA: Time-resolved immunofluorometric assay; CgA : chromogranin A; sAA : salivary α-amylase; TEA: total esterase activity; BChE: butyrylcholinesterase; Lip: lipase; Hp: haptoglobin; ADA: adenosine deaminase; CUPRAC: cupric reducing antioxidant capacity; FRAS: ferric reducing ability of saliva; UA: uric acid; AOPP: advanced oxidation protein products; Pox-Act: hydrogen peroxide; d-ROMs: reactive oxygen-derived compounds; ALT: alanine aminotransferase; AST: aspartate aminotransferase; ALP: alkalin phosphatase; GGT: γ-glutamil transferase; LDH: lactate dehydrogenase; CK: creatin kinase. [a] Beckman Coulter Inc., Fullerton, CA, USA. [b] Diazyme Laboratories, Poway, CA, USA. [c] Spinreact, Barcelona, Spain.
